# Estimability of migration survival rates from integrated breeding and winter capture–recapture data

**DOI:** 10.1002/ece3.4826

**Published:** 2019-02-05

**Authors:** Clark S. Rushing

**Affiliations:** ^1^ Department of Wildland Resources and the Ecology Center Utah State University Logan Utah; ^2^ Smithsonian Conservation Biology Institute Migratory Bird Center Washington District of Columbia

**Keywords:** Bayesian, Cormack‐Jolly‐Seber, demography, integrated population model, mark–recapture, migration, migratory connectivity, survival

## Abstract

Long‐distance migration is a common phenomenon across the animal kingdom but the scale of annual migratory movements has made it difficult for researchers to estimate survival rates during these periods of the annual cycle. Estimating migration survival is particularly challenging for small‐bodied species that cannot carry satellite tags, a group that includes the vast majority of migratory species. When capture–recapture data are available for linked breeding and non‐breeding populations, estimation of overall migration survival is possible but current methods do not allow separate estimation of spring and autumn survival rates. Recent development of a Bayesian integrated survival model has provided a method to separately estimate the latent spring and autumn survival rates using capture–recapture data, though the accuracy and precision of these estimates has not been formally tested. Here, I used simulated data to explore the estimability of migration survival rates using this model. Under a variety of biologically realistic scenarios, I demonstrate that spring and autumn migration survival can be estimated from the integrated survival model, though estimates are biased toward the overall migration survival probability. The direction and magnitude of this bias are influenced by the relative difference in spring and autumn survival rates as well as the degree of annual variation in these rates. The inclusion of covariates can improve the model's performance, especially when annual variation in migration survival rates is low. Migration survival rates can be estimated from relatively short time series (4–5 years), but bias and precision of estimates are improved when longer time series (10–12 years) are available. The ability to estimate seasonal survival rates of small, migratory organisms opens the door to advancing our understanding of the ecology and conservation of these species. Application of this method will enable researchers to better understand when mortality occurs across the annual cycle and how the migratory periods contribute to population dynamics. Integrating summer and winter capture data requires knowledge of the migratory connectivity of sampled populations and therefore efforts to simultaneously collect both survival and tracking data should be a high priority, especially for species of conservation concern.

## 1 INTRODUCTION

Seasonal migratory movements between breeding and non‐breeding areas are common phenomena across the animal kingdom (Alerstam, Hedenström, & Åkesson, 2003). These movements, which can range in scale from tens of meters to thousands of kilometers, induce complexities on the demographic processes that shape population dynamics of migratory species. Theoretical and empirical studies have demonstrated that not only can migratory species experience limiting factors during any stage of the annual cycle (e.g., breeding, winter, migration; Sherry & Holmes, 1996; Sutherland, 1996), but also that environmental and demographic processes can interact across periods (Marra, Cohen, Loss, Rutter, & Tonra, 2015). As a result, understanding the factors that limit and regulate dynamics of migratory species requires population models that can accommodate processes operating across the full annual cycle (Hostetler, Sillett, & Marra, 2015).

Full‐annual‐cycle models are a broad class of population models that include events occurring during both the breeding and non‐breeding periods (Hostetler et al., 2015). In recent years, development of full‐annual‐cycle models, driven in large part by the need inform conservation planning for declining migratory species, has increased our understanding breeding vs. winter population limitation (Robinson et al., 2016; Rushing, Ryder, & Marra, 2016; Taylor, 2017). Most full‐annual‐cycle models, however, have either focused only on events occurring during the stationary breeding and winter periods or have lumped the migration and winter periods into a single “non‐breeding” period (e.g., Wilson, LaDeau, Tøttrup, & Marra, 2011). As a result, the impact of the spring and autumn migration on the dynamics of migratory species remains poorly understood.

The primary obstacle to accounting for the migratory periods in full‐annual‐cycle models is the inability to quantify survival during these periods. For large species (>~100 g), the development of miniaturized satellite tags has revolutionized our ability to track migratory movements and mortality rates during these periods (e.g., Klaassen et al., 2014). Most species, however, are too small to directly track during migration (Bridge et al., 2011) and therefore survival during these periods can only be estimated from indirect (e.g., capture–mark–recapture) methods. In a seminal paper, Sillett and Holmes (2002) used capture–recapture data from linked breeding and winter populations of Black‐throated Blue Warblers (*Setophaga caerulescens*) to estimate overall migration survival (i.e., cumulative spring and autumn survival) and demonstrate that the majority of annual mortality in this species occurs during these periods. Subsequent application of this approach to several other migratory passerines (Paxton, Durst, Sogge, Koronkiewicz, & Paxton, 2017; Rockwell et al., 2017) has corroborated results from Sillett and Holmes (2002) showing the highest seasonal mortality during migration. However, the method used by Sillett and Holmes (2002) was not developed to separately estimate survival during spring and autumn migration. This limitation has prevented a full understanding of when mortality occurs across the annual cycle as well as how the survival during the migratory periods influences population dynamics.

Recently, Rushing et al. (2017) developed a novel integrated population model (IPM) to separately estimate spring and autumn migration survival. The core of this model is an integrated survival model that uses capture–mark–recapture data collected during both the breeding and winter periods. By integrating the two data sets, it is possible to estimate the latent spring and autumn survival rates (Rushing et al., 2017), though the accuracy and precision of these estimates has not been formally tested. Here, I used simulated data to explore the identifiability and estimability of migration survival rates using the integrated survival model. Under a variety of biologically realistic scenarios, I demonstrate that spring and autumn migration survival are identifiable and can be estimated from the integrated capture–recapture model. I also show that the inclusion of covariates can improve the model's performance compared to the use of capture data alone. These results open the door for full‐annual‐cycle population models to provide deeper understanding of the ecology of migratory species.

## 2 MATERIALS AND METHODS

**Figure 1:**
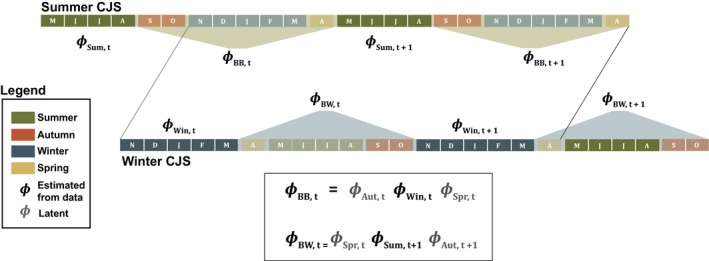
Conceptual diagram of the integrated survival model

The models described here assume a simple migratory annual cycle, with two stationary periods separated by distinct migratory stages. In the remainder of the paper, I refer to the stationary periods as “breeding” and “winter” and to the migratory periods as “spring” and “autumn” (Figure 1). For all simulations, I assume a 4 month breeding season, 2 month autumn migration, 5 month winter period, and 1 month spring migration.

To infer survival during spring and autumn, the integrated model requires data sufficient to estimate survival within and between each stationary period. In practice, these estimates could come from a variety of data types and model frameworks but here I assume standard capture–mark–recapture data appropriate for estimating apparent survival using the basic Cormack‐Jolly‐Seber (CJS) model (Lebreton, Burnham, Clobert, & Anderson, 1992). I further assume that sampling within each stationary period takes place at the beginning and again at the end of each season, allowing one to estimate both within‐ and between‐season survival probabilities (Figure 1). In the remainder of the paper, I refer to survival between breeding periods as *ϕ*_BB_ and survival between winter periods as *ϕ*_BW_.

As illustrated in Figure 1, *ϕ*_BB_ and *ϕ*_BW_ contain information about the latent autumn and spring survival rates. By integrating the breeding and winter CJS models in a unified analysis, the between‐season survival rates can be parameterized in terms of the underlying seasonal survival parameters. Specifically,

(1) $$\phi_{\mathrm{BB},t}=\phi_{\mathrm{Aut},t}\phi_{\mathrm{Win},t}\phi_{\mathrm{Spr},t}$$

(2) $$\phi_{\mathrm{BW},t}=\phi_{\mathrm{Spr},t}\phi_{\mathrm{Sum},t+1}\phi_{\mathrm{Aut},t+1}$$

where *ϕ*_Sum,*t*_ and *ϕ*_Win,*t*_ are the summer and winter survival probabilities estimated from the capture–recapture data and *ϕ*_Aut,*t*_ and *ϕ*_Spr,*t*_ are the latent autumn and spring survival rates. When repeated over multiple years of sampling, Equations (1) and (2) provide a system of equations that can be parameterized in terms of the latent survival rates.

In this paper, I used simulated data to assess the identifiability and estimability of the latent spring and autumn survival rates. In CMR models, parameter identifiability can be assessed by simulating capture histories for a very large number of individuals and then quantifying the bias of parameter estimates from the model (Gimenez, Viallefont, Catchpole, Choquet, & Morgan, 2004). With large sample sizes, the observed frequency of encounter histories should be equal to the expected frequency (i.e., no sampling error), and therefore bias in the estimated parameters indicates a lack intrinsic identifiability. In some cases, parameters may technically be identifiable but may nonetheless not be estimable given the data at hand (Auger‐Méthé et al., 2016). To investigate estimability of the latent survival rates, I simulated CMR data with sample sizes more typical of CMR studies and assessed the bias and precision of estimates based on these data.

### 2.1 Simulating survival data

For each simulation, I generated data consistent with typical capture–mark–recapture (CMR) sampling protocols. All simulations consisted of two CMR data sets collected during both summer and winter. For tests of identifiability, I simulated data with 10,000 new individuals captured in each year. This number was chosen to be large enough that parameter estimates were not influenced by sampling error (Gimenez et al., 2004). For tests of estimability, I assumed 75 new individuals captured each year, a sample size more typical of many CMR studies. Mean monthly survival probabilities during summer, winter, and autumn were held constant across all simulations (*μ*_Sum_ = 0.97, *μ*_Win_ = 0.98, *μ*_Aut_ = 0.90). Mean monthly spring survival (*μ*_Spr_) varied across simulations (described below). These monthly survival rates were chosen to produce biologically realistic annual survival rates for a small, migratory songbird (~0.43–0.58). Each simulation consisted of the following steps:

1. Determine mean spring migration survival

For each simulation, *μ*_Spr_ was determined as:

(3) $$\mu_{\mathrm{Spr}}=\Delta \times \mu_{\mathrm{Aut}}$$

where ∆ is the relative difference between *μ*_Spr_ and *μ*_Aut_.

- Simulate realized autumn/spring survival probabilities

For each year *t*, realized monthly survival probability in autumn and spring were simulated as:

(4) $$\text{logit}\left(\phi_{j,t}\right)=\text{logit}\left(\mu_{j}\right)+\beta_{j}X_{j,t}+\varepsilon_{j,t},\;\varepsilon_{j,t}∼\text{MV}\left(0,\Sigma \right)$$

$$\Sigma =\left(\begin{array}{cc} \sigma_{\mathrm{Aut}}^{2} & \rho \sqrt{\sigma_{\mathrm{Aut}}^{2}\sigma_{\mathrm{Spr}}^{2}}\\ \rho \sqrt{\sigma_{\mathrm{Aut}}^{2}\sigma_{\mathrm{Spr}}^{2}} & \sigma_{\mathrm{Spr}}^{2} \end{array}\right)$$

where *ϕ*_*j*,*t*_ is the realized monthly survival rate for season *j* (autumn or spring), *β*_*j*_ is the effect of covariate *X*_*j*,*t*_ on *ϕ*_*j*,*t*_, *Σ* is the variance–covariance matrix describing annual variation in spring and autumn migration, $\sigma_{\mathrm{Aut}}^{2}$ and $\sigma_{\mathrm{Spr}}^{2}$ are the annual variances of autumn and spring survival, and *ρ* is the correlation between autumn and spring survival in a given year. Parameterizing the yearly spring and autumn survival rates in this way made it possible to independently vary the annual variance in and correlation between spring and autumn migration.

- Generate Φ matrix

The monthly *ϕ*_*j*,*t*_ rates were converted into survival across the entire season by raising each to the appropriate number of months. The seasonal survival rates were then arranged in a matrix Φ containing the survival rates across all 48 seasons (12 years × 4 seasons/year):

$$\Phi =\left[0.97^{4},{\phi_{\mathrm{Aut},1}}^{2},0.98^{5},\phi_{\mathrm{Spr},1},\ldots ,0.97^{4},{\phi_{\mathrm{Aut},12}}^{2},0.98^{5},\phi_{\mathrm{Spr},12}\right]$$

- Simulate summer/winter survival histories

Survival histories were simulated for individuals in both the summer and winter populations using the occasion‐specific survival probabilities in Φ. Conditional on first capture, survival of individual *i* across all subsequent seasons was modeled as:

(5) $$z_{i,j}∼\text{Bernoulli}\left(z_{i,j-1}\Phi_{j-1}\right)$$

where *z*_*i*,*j*_ is the true state (0 = dead, 1 = alive) of individual *i* during season *j*, and *Φ*_*j*−1_ is the survival probability from season *j*−1 to season *j*. Note that although summer and winter survival histories were generated independently (i.e., did not share any individuals), individuals in both data sets shared the same survival rates during each occasion.

- Simulate capture histories

To account for imperfect detection during each sampling period, individual capture histories were generated based on each individual's true state at sampling occasion *k* and a season‐specific monthly recapture probability *p*_*j*_:

(6) $$y_{i,j,k}∼\text{Bernoulli}\left(z_{i,j}p_{j}\right)$$

where *y*_*i*,*j*,*k*_ is the observed state (0 = not recaptured, 1 = recaptured) during season *j* on occasion *k* (beginning or end of season). For all simulations, *p*_Sum_ = 0.6 and *p*_Win_ = 0.4.

### 2.2 Simulation scenarios

Equations (3), [Link], (5) contain several parameters that may influence identifiability and estimability of the latent spring and autumn survival rates. To quantify the effects of these factors on model performance, I generated capture histories under a range of simulation scenarios:

*“Basic” model:* To understand the performance of the integrated CJS model in instances where no additional information is available (e.g., covariates), I simulated 12 years of CMR data following steps 1–5 while manipulating three parameters: Δ, $\sigma_{j}^{2}$, and *ρ*. In the remainder of the paper, I refer to this as the “basic” model. For each parameter, data were simulated under three levels corresponding to low, medium, and high values. To examine the influence of the relative difference between *μ*_Spr_ and *μ*_Aut_, data were generated assuming Δ = 1, 0.875, and 0.75. Because *μ*_Aut_ was held constant at 0.9 in all simulations, these scenarios correspond to *μ*_Spr_ = 0.9, 0.78, and 0.675. To examine the effect of annual variation in migration survival on identifiability and estimability, data were generated assuming $\sigma_{j}^{2}$ = 0.02, 0.25, and 0.50. To minimize the total number of simulation scenarios, I assumed that $\sigma_{\mathrm{Aut}}^{2}=\sigma_{\mathrm{Spr}}^{2}$. To examine the effect of the correlation between *ϕ*_Spr*,t*_ and *ϕ*_*A*ut,*t*_, data were generated assuming *ρ* = 0, 0.4, and 0.8. In all cases, parameter values were chosen to produce biologically realistic survival rates. For all “basic” model simulations, *β*_*j*_ in Eq. (4) was fixed at 0. The three parameters were varied in a factorial design, resulting in 3^3^ = 27 simulation scenarios.
*Covariate model:* To investigate whether including covariates improves estimation of the latent migration survival rates, I conducted additional simulations with a range of *β*_*j*_ values for both spring and autumn (0, 0.5, 1.0) and *σ* ^2^ values (0.02, 0.25, 0.50). Annual values for each covariate *X*_*j*,*t*_ were simulated from a normal distribution with mean 0 and standard deviation of 1. As for the basic model, these three parameters (*β*_Aut_, *β*_Spr_, and *σ* ^2^) were varied in a factorial design resulting in 27 scenarios. All covariate models included 12 years of CMR data and assumed Δ = 0.75 and *ρ* = 0. I did not conduct identifiability simulations for the covariate model because if the parameters are identifiable under the basic model, than they should also be identifiable with the addition of covariates.
*Number of years:* Estimability of survival rates in CJS models is influenced by the number of years of capture–recapture data included in the analysis (Pollock, Nichols, Brownie, & Hines, 1990). To investigate how study length influences estimability of migration survival rates, I conducted additional simulations of the “basic” model with 4–11 years of data, resulting in 8 scenarios. For all study length simulations, Δ = 0.75, *σ* ^2^ = 0.25, and *ρ* = 0.

### 2.3 Model fit

For each scenario, I simulated a single data set for the identifiability tests and 250 data sets for the estimability tests. I estimated the joint likelihood of the model using JAGS version 3.3.0 (Plummer, 2012) called from program R version 3.3.1 (R Core Team, 2016) with package jagsUI version 1.4.2 (Kellner, 2016). Breeding and winter monthly survival rates were given uninformative Uniform(0,1) priors and beta coefficients in the covariate models were given uninformative Normal(0,100) priors. The monthly spring and autumn survival probabilities were given weakly informative Beta(3,2) priors. Initial model testing indicated that this prior improved mixing of the chains compared to an uninformative uniform prior (effective sample sizes were ~4× higher under the Beta prior) but did not meaningfully influence posterior means (on average, posterior means differed by <0.01 under the Uniform vs. Beta priors). For all models, I ran three chains for 50,000 iterations each after an adaptation phase of 5,000 iterations and discarding the first 10,000 iterations as burn‐in. To reduce autocorrelation in the chains, I saved every 10th iteration. Convergence was confirmed through Rhat values and visual inspections of trace plots.

### 2.4 Model evaluation

Model performance was measured using five metrics. To measure identifiability of the mean survival rates (*μ*_Spr_ and *μ*_Aut_) under the “basic” model, I measured relative bias under each scenario as $\left({\hat{\mu }}_{i,j}-\mu_{j}\right)/\mu_{j}$, where ${\hat{\mu }}_{j}$ is the estimated mean survival rate for season *j*, and *μ*_*j*_ is the true mean survival rate. Parameters were considered identifiable if the relative bias was >−0.01 and <0.01.

For each of the estimability scenarios, mean relative bias and root mean square error (RMSE) of *μ*_Spr_ and *μ*_Aut_ were measured as:

(7) $${\text{Bias}}_{j}=\frac{\sum_{i=1}^{250}\left({\hat{\mu }}_{i,j}-\mu_{j}\right)/\mu_{j}}{250}$$

(8) $${\text{RMSE}}_{j}=\sqrt{\frac{\sum_{i=1}^{250}{\left({\hat{\mu }}_{i,j}-\mu_{j}\right)}^{2}}{250}}$$

In some applications, researchers may also be interested in determining which season has the lowest survival. For simulations in which Δ < 1, I also estimated the proportion of simulations in which ${\hat{\mu }}_{\mathrm{Spr}}<{\hat{\mu }}_{\mathrm{Aut}}$. This metric provides an estimate of the power of the model to correctly infer which season has the lowest survival.

For the annual estimates (*ϕ*_Aut,*t*_ and *ϕ*_Spr,*t*_), performance was measured as the mean correlation between the estimated and true values:

(9) $$r_{j}=\frac{\sum_{i=1}^{250}\text{cor}({\hat{\phi }}_{i,j,t},\phi_{j,t})}{250}$$

where ${\hat{\phi }}_{i,j,t}$ is the estimated survival for season *j* in year *t* in simulation *i*.

## 3 RESULTS

**Figure 2:**
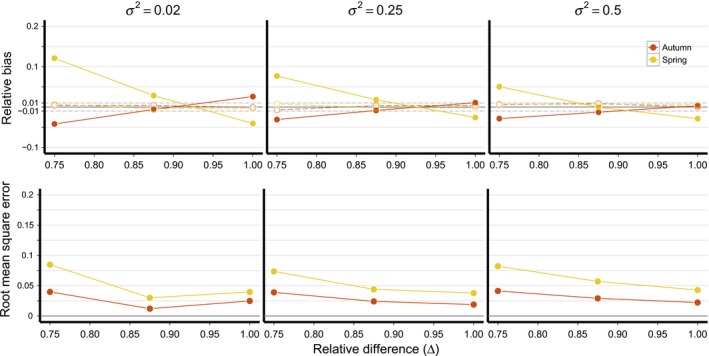
Relative bias and root mean square error of mean monthly survival estimates for spring and autumn migration as a function of the relative difference in survival between the two seasons (∆) and annual variation in survival rates (*σ* ^2^). Relative bias of identifiability models are indicated by open circles/dashed lines and relative bias of estimability models are indicated by filled circles/solid lines. In all simulations shown, *ρ* = 0

For the “basic” model, the relative bias of mean monthly spring and autumn survival (${\hat{\mu }}_{\mathrm{Spr}}$ and ${\hat{\mu }}_{\mathrm{Aut}}$) was <0.01 for all parameter combinations, indicating that these parameters are identifiable under all simulated scenarios (Figure 2, Supporting Information Table S1). However, under more realistic sample sizes ${\hat{\mu }}_{\mathrm{Spr}}$ and ${\hat{\mu }}_{\mathrm{Aut}}$ were biased toward the overall mean migration survival rate (i.e., $\mu_{\mathrm{Aut}}^{2}\times \mu_{\mathrm{Spr}}$). The magnitude of both bias and root mean square error (RMSE) were proportional to the relative difference between the seasonal survival rates (Δ) and the magnitude of annual variation in survival rates (*σ* ^2^; Figure 2). When mean monthly survival in spring and autumn were equal (Δ = 1), was biased on average by −2.92% while ${\hat{\mu }}_{\mathrm{Aut}}$ was biased by 1.27%. Note that when monthly survival rates are equal, survival across the entire 2 month autumn period is lower than survival during the 1 month spring period, resulting in negative bias in ${\hat{\mu }}_{\mathrm{Spr}}$ and positive bias in ${\hat{\mu }}_{\mathrm{Aut}}$. When Δ = 0.75, the direction of bias switched and the magnitude of bias in ${\hat{\mu }}_{\mathrm{Spr}}$ increased to 8.62% and to −3.2% for ${\hat{\mu }}_{\mathrm{Aut}}$ (Figure 2).

The degree of bias in ${\hat{\mu }}_{\mathrm{Spr}}$ and ${\hat{\mu }}_{\mathrm{Aut}}$ was inversely related to *σ* ^2^ (Figure 2). Assuming Δ = 0.75, bias in ${\hat{\mu }}_{\mathrm{Spr}}$ was 11.98% and bias in ${\hat{\mu }}_{\mathrm{Aut}}$ was −4.11% when *σ* ^2^ = 0.02. In contrast, when *σ* ^2^ = 0.5, bias in ${\hat{\mu }}_{\mathrm{Spr}}$ declined to 6.18% and bias in ${\hat{\mu }}_{\mathrm{Aut}}$ declined to −2.74%. Despite the sources of bias in the basic model, power to detect the direction of survival differences (i.e., whether survival was lower in spring or autumn) was high (range = 87%–100%). Thus, the basic model was generally successful at determining which period had lower survival but tended to underestimate the difference between the two periods. Estimates of ${\hat{\mu }}_{\mathrm{Spr}}$ and ${\hat{\mu }}_{\mathrm{Aut}}$ were not influenced by correlation between spring and autumn migration (*ρ*; Supporting Information Figures S1 and S2).

**Figure 3:**
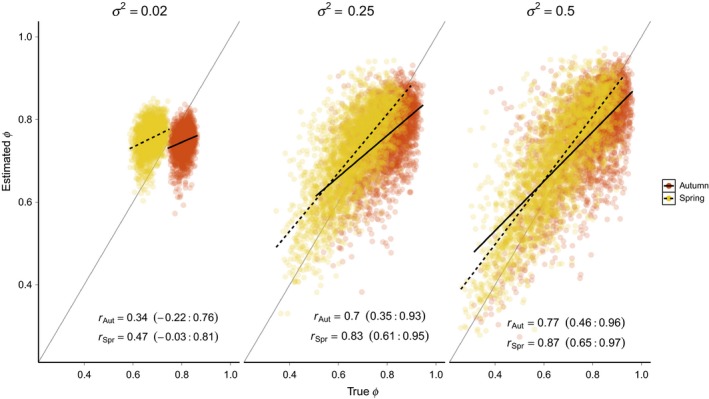
Correlation between estimated and true spring and autumn survival rates under the basic model. For all simulations shown, ∆ = 0.75 and *ρ* = 0. Points show estimates of *ϕ*_*j,t*_ from all 250 simulations in each scenario. Solid and dashed black lines show the mean correlation for each season and the solid gray line indicates 1:1 correspondence between estimated and true survival. Values in parentheses are the 95% credible interval of the *r* estimates

In all “basic” model scenarios, estimates of spring and autumn survival were positively correlated with true survival but the magnitude of the correlation was strongly affected by *σ* ^2^ (Figure 3). When spring and autumn survival showed little annual variation (*σ* ^2^ = 0.02), the correlation was small and non‐significant (*r*_Spr_ = 0.47, 95% credible interval = −0.03 to 0.81; *r*_Aut_ = 0.34, −0.22 to 0.76). However, as annual variation increased, survival estimates were more strongly correlated with true survival (*σ* ^2^ = 0.25: *r*_Spr_ = 0.83, 0.61–0.95; *r*_Aut_ = 0.7, 0.35–0.93; *σ* ^2^ = 0.50: *r*_Spr_ = 0.87, 0.65–0.97; *r*_Aut_ = 0.77, 0.46–0.96). The correlations between true and estimated survival were inversely related to Δ but were not influenced by *ρ* (Supporting Information Figure S3).

**Figure 4:**
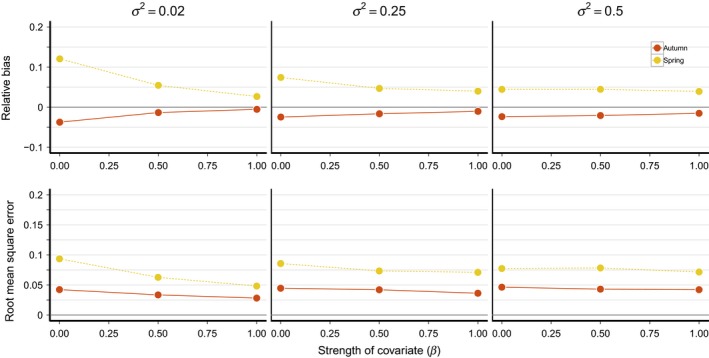
Relative bias and root mean square error of mean monthly survival estimates for spring and autumn migration as a function of covariate effect size (*β*) and annual variation in survival rates (*σ* ^2^). The *x*‐axis refers to the simulated value of both *β*_Spr_ and *β*_Aut_. In all simulations shown, Δ = 0.75 and *ρ* = 0

**Figure 5:**
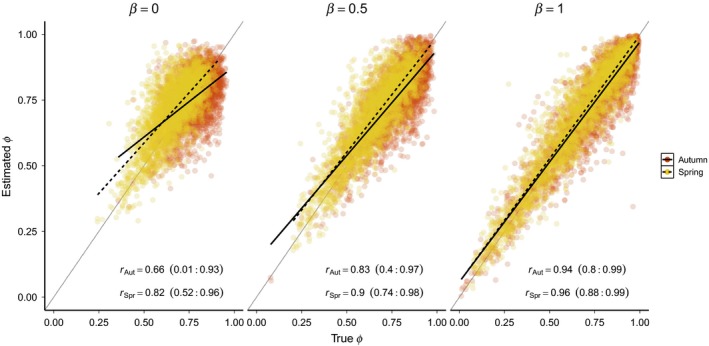
Correlation between estimated and true spring and autumn survival rates under the covariate models. In each panel, *β* refers to the simulated value of both *β*_Spr_ and *β*_Aut_. For all simulations shown, *σ* ^2^ = 0.25. Points show estimates of *ϕ*_*j,t*_ from all 250 simulations in each scenario. Solid and dashed black lines show the mean correlation for each season and the solid gray line indicates 1:1 correspondence between estimated and true survival. Values in parentheses are the 95% credible interval of the *r* estimates

Including covariates in the model improved estimation of migration survival rates compared to the basic model, though the degree of improvement depended on *σ* ^2^. When *σ* ^2^ = 0.02, including covariates in the model greatly reduced both bias and RMSE (Figure 4). In this scenario, including covariates with a strong effect (*β* = 1) reduced bias in ${\hat{\mu }}_{\mathrm{Spr}}$ by 78% (3% when *β* = 1 vs. 12% when *β* = 0) and by 86% (−1% vs. −4%), despite a large relative difference between the two seasons (Δ* *= 0.75). In contrast, when *σ* ^2^ = 0.5, the effect of covariates was much smaller (12% and 35% decreases in bias of ${\hat{\mu }}_{\mathrm{Spr}}$ and ${\hat{\mu }}_{\mathrm{Aut}}$, respectively). RMSE was similarly decreased through the inclusion of covariates and correlation between true and estimated survival was increased. For example, when strong covariates were included on both autumn and spring survival, *r*_Aut_ increased to 0.93 (95% credible interval = 0.77–0.99) and *r*_Spr_ increased to 0.95 (95% credible interval = 0.86–0.99; Figure 5). As in the basic model, power to detect the direction of survival differences was high when covariates were included in the model (range = 90–100%).

**Figure 6:**
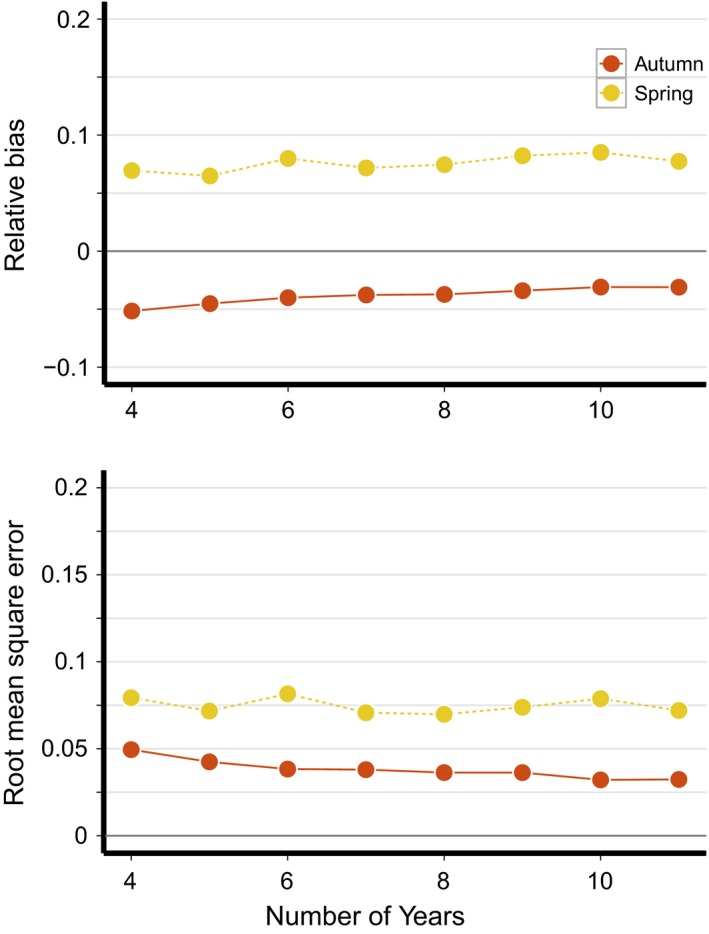
Relative bias and root mean square error of mean monthly survival estimates for spring and autumn migration as a function of study length. In all simulations shown, Δ = 0.75, *σ* ^2^ = 0.25, and *ρ* = 0

**Figure 7:**
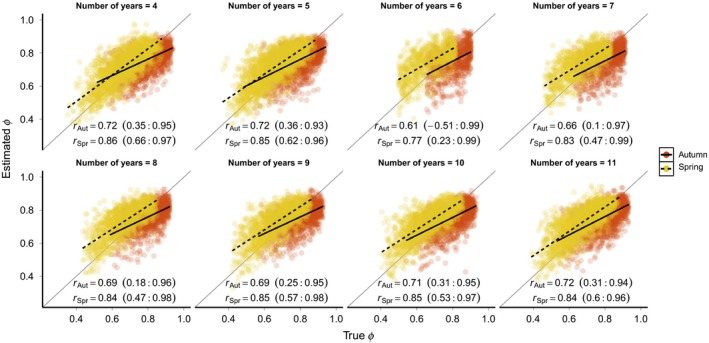
Correlation between estimated and true spring and autumn survival rates under different simulated study lengths. For all scenarios shown, Δ = 0.75, *σ* ^2^ = 0.25, and *ρ* = 0. Points show estimates of *ϕ*_*j,t*_ from all 250 simulations in each scenario. Solid and dashed black lines show the mean correlation between true and estimated survival for each season and the solid gray line indicates 1:1 correspondence. Values in parentheses are the 95% credible interval of the r estimates

Both bias and RMSE of ${\hat{\mu }}_{\mathrm{Aut}}$ tended to decrease as additional years of capture–recapture data were included in the analysis (Figure 6), but reached an asymptote with ~10 years of data. Interestingly, neither bias or RMSE of ${\hat{\mu }}_{\mathrm{Spr}}$ estimates were influenced by the number of years of data. The mean correlation between the true and estimated yearly survival rates tended to increase with additional years of data when the number of years was less than 6 but beyond 6–7 years of data there was no further increase in the mean *r* for either season. However, longer time frames greatly improved the precision of the *r* estimates, as evident from the decreasing width of the *r* credible intervals as the number of years increased (Figure 7).

## 4 DISCUSSION

The twice‐annual migrations made by billions of individual organisms each year are among the most fascinating phenomena in the natural world. These movements have important implications for the population dynamics and conservation of migratory species but have proven difficult to study in most species. Using simulated data, I demonstrate that the integrated survival model developed by Rushing et al. (2017) is capable of estimating latent spring and autumn survival probabilities from capture–recapture data under certain conditions.

Tests of identifiability indicate that mean monthly spring and autumn survival rates are identifiable using the integrated survival model. However, estimates of these rates were biased in simulations that assumed sample sizes more typical of many CMR studies (75 new individuals released at each occasion). In particular, estimates of *μ*_*Spr*_ and *μ*_*Aut*_ were biased toward the overall “migration” survival, suggesting that the model had trouble pulling apart the seasonal survival rates without larger sample sizes. The degree of bias was positively related to the relative difference between spring and autumn survival and negatively related to the amount of annual variation in these survival rates. Thus, bias in the basic model was lowest when the difference between spring and autumn survival was small and when annual variation was high. Lower bias with increasing year‐to‐year variation is likely the result of smaller ranges of plausible combinations of spring and autumn survival in the time series defined by Eqs. (1) and (2). The correlation between estimated and true migration survival rates (*r*) was also influenced by *σ* ^2^, with higher correlations occurring when annual variation was high. Given the bias in the mean survival rates, users should interpret their results carefully and are encouraged to analyze simulated data based on their actual sample sizes and estimated parameters as a post hoc assessment of potential bias in their parameter estimates. Despite bias toward the overall migration survival rate, the model had high power to detect which season had lower survival.

Including covariates in the model improved estimation of spring and autumn migration rates. When annual variation in these rates was small, the additional information provided by the covariates greatly reduced bias and RMSE and increased the correlation between estimated and true survival compared to the basic model, even when covariates had only a moderate effect (*β* = 0.5; Supporting Information Figures S4–S6). However, when annual variation in spring and autumn survival rates was high, including covariates resulted in only small improvements to parameter estimates, likely because the plausible combinations of spring and autumn survival were already reduced by the year‐to‐year variation. Interestingly, covariates have little effect on the estimation of survival rates during the opposite migratory period (Supporting Information Figure S6), suggesting that model performance will be best when covariates are included for both spring and autumn migration.

For most species, researchers may have little a priori knowledge about the demographic or environmental processes that influence migration survival. In these cases, it may be useful to identify processes known to influence annual survival and test these as covariates on spring and/or autumn migration. For example, Sillett, Holmes, and Sherry (2000) found that El Niño/La Niña cycles have a strong influence on annual survival of Black‐throated Blue Warblers wintering in Jamaica. Subsequent analysis of these data using the framework presented here indicated that the El Niño/La Niña effects primarily influence spring migration survival rather than survival during the stationary winter or breeding periods (C. S. Rushing and T. S. Sillett, unpublished). Because covariates of annual survival are known for many species, this may be a useful approach for improving estimates of migration survival in many species.

Currently, only one published study has used this integrated survival model to estimate the latent spring and autumn survival rates from capture–recapture data. Using a modification of the basic model presented here, Rushing et al. (2017) found that apparent spring migration survival of Wood Thrush (*Hylocichla mustelina*) was ~5% and 50% lower than autumn survival for adults and juveniles, respectively. Based on the results presented in this paper, we conclude that the direction of these differences (*μ*_Spr_ < *μ*_Aut_) is likely correct but that the magnitudes of the differences were likely underestimated.

In addition to the assumptions of conventional CJS models, the integrated survival model assumes that individuals in each population have the same seasonal survival rates. Thus, although it is not necessary to sample the same individuals in each season, the integrated model does require data from linked breeding and winter populations. In reality, most breeding CMR data will contain individuals that winter in different locations and vice versa for winter CMR data. The degree to which individuals maintain geographic proximity across the annual cycle, termed migratory connectivity (Webster, Marra, Haig, Bensch, & Holmes, 2002), as well as the degree to which seasonal survival rates vary among populations could produce complex forms of heterogeneity that were not included in the simulations presented here. The influence of migratory connectivity on estimation of seasonal survival rates requires additional study, though due to the complexity of possible patterns and strengths of migratory connectivity (Cohen et al., 2018), this topic is beyond the scope of this paper. Until the effects of migratory connectivity are better understood, users of this method should at least provide evidence that their data comes from linked breeding and winter populations (Rushing et al., 2017).

The ability to estimate seasonal survival rates of small, migratory organisms opens the door to advancing our understanding of these species. At present, application of this method is likely restricted to a few well‐studied species that have adequate survival data from linked populations. Future efforts focused on quantifying migratory connectivity and collecting mark–recapture data, especially from wintering populations, are urgently needed for many other species, especially those of conservation concern. Collection of those data, along with further development of integrated models for estimating seasonal survival and population dynamics, will provide even deeper insights into the ecology and conservation of migratory species.

## CONFLICT OF INTEREST

None declared.

## AUTHOR CONTRIBUTION

All authors conceived of and conducted the analyses, drafted the manuscript, and contributed equally to revisions.

## Data Availability

Upon acceptance, all code necessary for simulating data, fitting models, and generating figures will be available on the author's personal github page (http://github.com/crushing05/FACsim) and can be installed and loaded as an R package using the commands install.packages(“devtools”); devtools::install_github(‘crushing05/FACsim).
