# Supplementary material for: Estimability of migration survival rates from integrated breeding and winter capture–recapture data
Source: Ecol Evol. 2019 Feb 5;9(2):849–58. doi: 10.1002/ece3.4826 (PMC6362449; doi:10.1002/ece3.4826)
Supplement: Supplementary file 1 [file ECE3-9-849-s001.pdf]

Appendix S1

Additional figures providing results for all simulation scenarios from Rushing, CS (in review) Estimability of migration survival rates from integrated breeding and winter capture-recapture data. *Methods in Ecology and Evolution*.

Table S1: Relative bias of spring and fall survival estimates for tests of identifiability under the 'basic' integrated survival model (no covariates). See

Simulation	Autumn bias	Spring bias	Δ	σ^2	ρ
1	-0.0007	-0.0040	1.000	0.02	0.0
2	0.0041	-0.0042	0.875	0.02	0.0
3	0.0040	0.0073	0.750	0.02	0.0
4	0.0039	-0.0023	1.000	0.25	0.0
5	0.0036	-0.0019	0.875	0.25	0.0
6	-0.0071	0.0080	0.750	0.25	0.0
7	-0.0002	0.0060	1.000	0.50	0.0
8	0.0087	0.0067	0.875	0.50	0.0
9	0.0059	0.0072	0.750	0.50	0.0
10	-0.0005	0.0030	1.000	0.02	0.4
11	0.0049	0.0008	0.875	0.02	0.4
12	0.0079	0.0054	0.750	0.02	0.4
13	-0.0007	0.0070	1.000	0.25	0.4
14	0.0028	0.0075	0.875	0.25	0.4
15	0.0069	-0.0046	0.750	0.25	0.4
16	0.0042	0.0043	1.000	0.50	0.4
17	0.0088	0.0020	0.875	0.50	0.4
18	-0.0055	0.0020	0.750	0.50	0.4
19	-0.0022	0.0045	1.000	0.02	0.8
20	-0.0001	-0.0037	0.875	0.02	0.8
21	0.0076	0.0005	0.750	0.02	0.8
22	-0.0003	0.0005	1.000	0.25	0.8
23	0.0060	-0.0011	0.875	0.25	0.8
24	0.0007	0.0063	0.750	0.25	0.8
25	0.0038	-0.0012	1.000	0.50	0.8
26	-0.0075	0.0014	0.875	0.50	0.8
27	-0.0011	0.0064	0.750	0.50	0.8

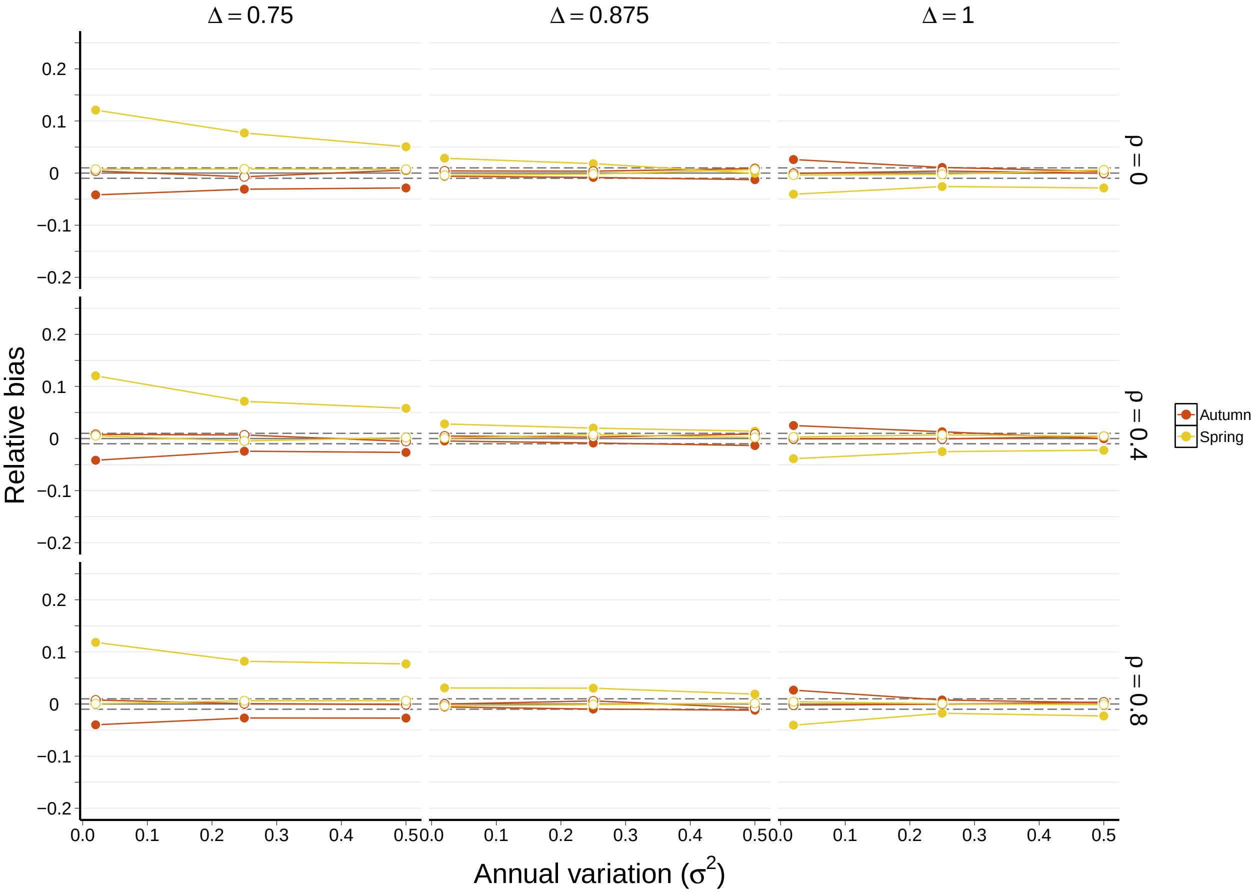

Figure S1: Relative bias of mean monthly survival estimates for spring and autumn migration as a function of annual variation in survival rates (σ^2), the relative difference in survival between the two seasons (Δ) and the correlation between spring and autumn survival (ρ). Relative bias of identifiability models are indicated by open circles/dashed lines and relative bias of estimability models are indicated by filled circles/solid lines.

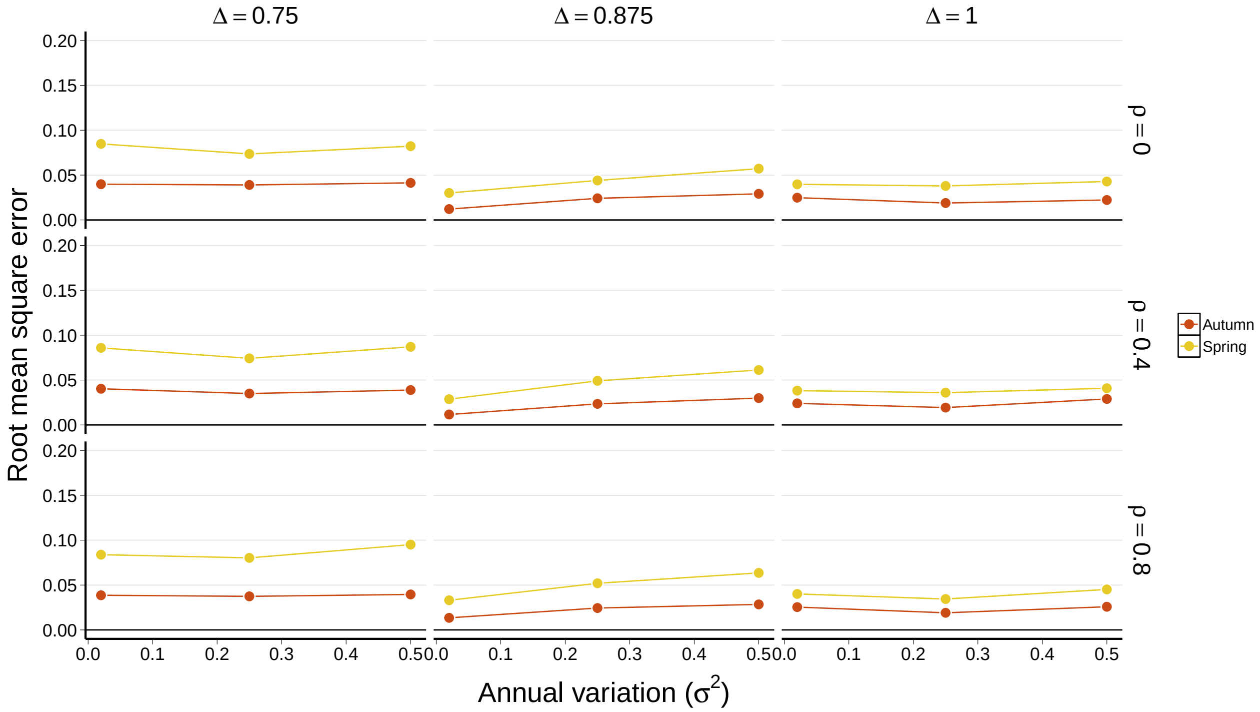

Figure S2: Root mean square error of mean monthly survival estimates for spring and autumn migration as a function of annual variation in survival rates (σ^2), the relative difference in survival between the two seasons (Δ) and the correlation between spring and autumn survival (ρ).

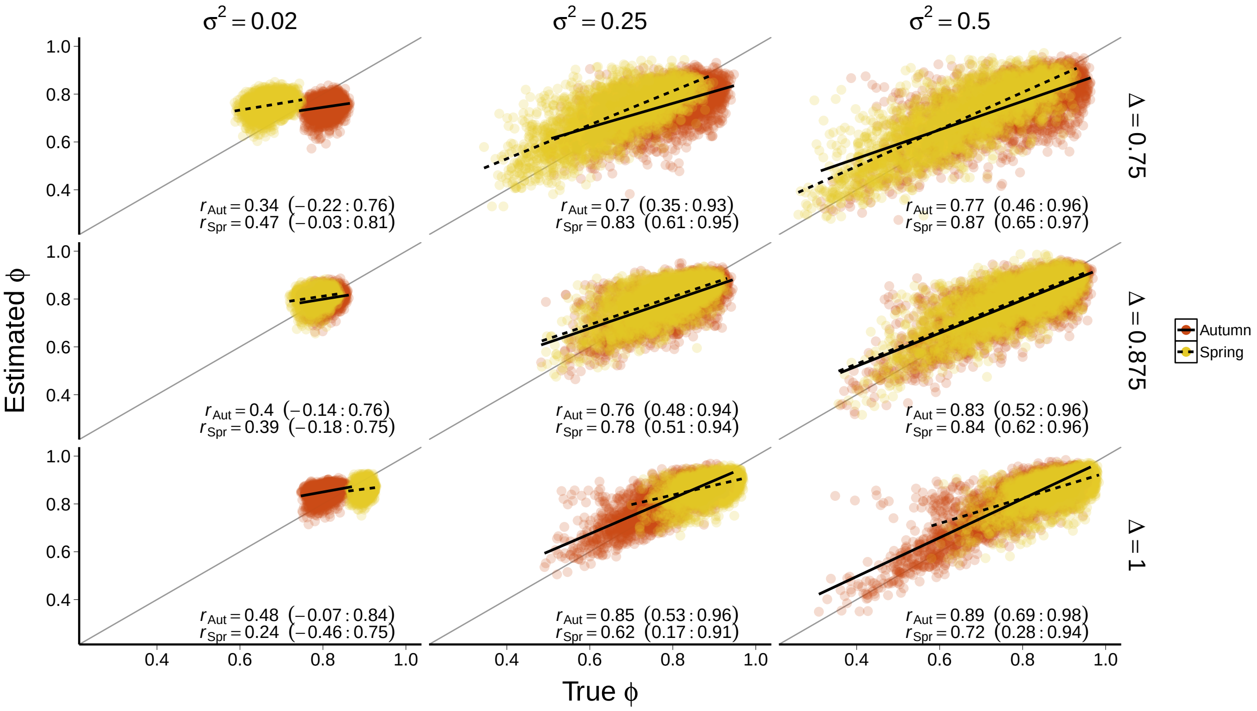

Figure S3: Correlation between estimated and true spring and autumn survival rates under the basic model. For all simulations shown, $\rho = 0$. Points show estimates of $\phi_{j,t}$ from all 250 simulations in each scenario. Solid and dashed black lines show the mean correlation for each season and the solid gray line indicates 1:1 correspondance between estimated and true survival. Values in parentheses are the 95% credible interval of the r estimates.

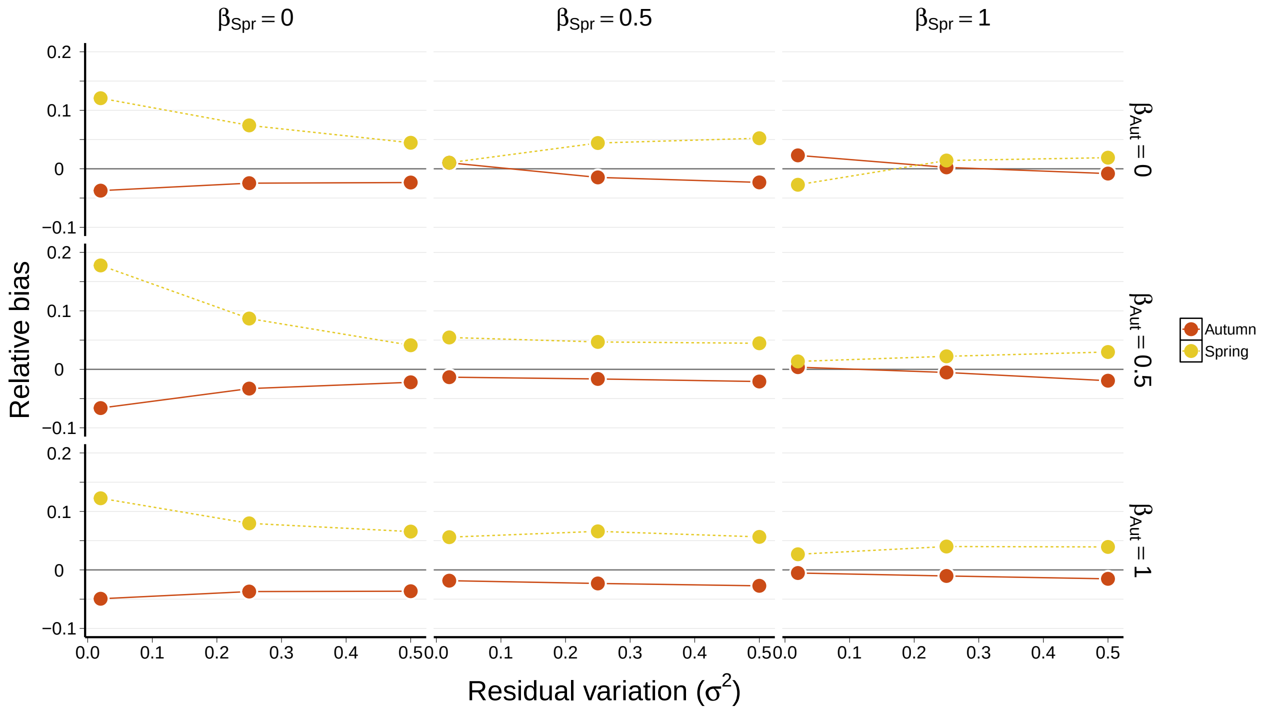

Figure S4: Relative bias of mean monthly survival estimates for spring and autumn migration as a function of covariate effect size (β) and annual variation in survival rates (σ^2). In all simulations shown, $\Delta = 0.75$ and $\rho = 0$.

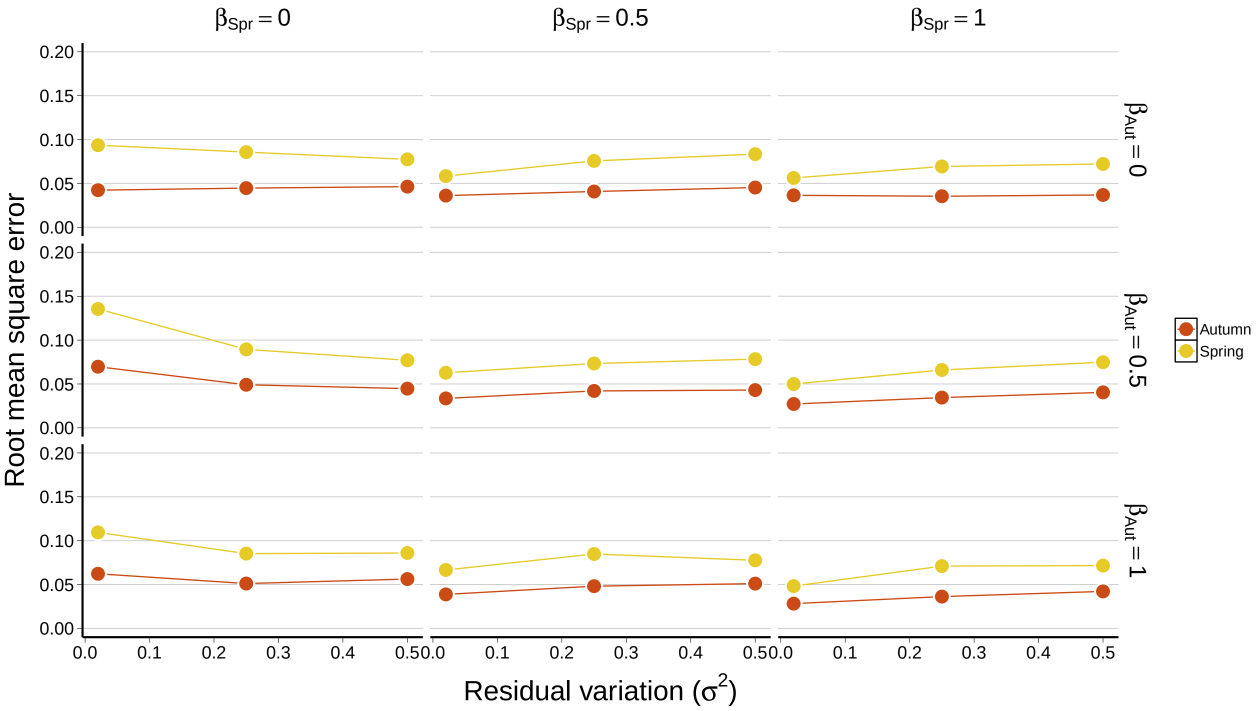

Figure S5: Root mean square error of mean monthly survival estimates for spring and autumn migration as a function of covariate effect size (β) and annual variation in survival rates (σ^2). In all simulations shown, $\Delta = 0.75$ and $\rho = 0$.

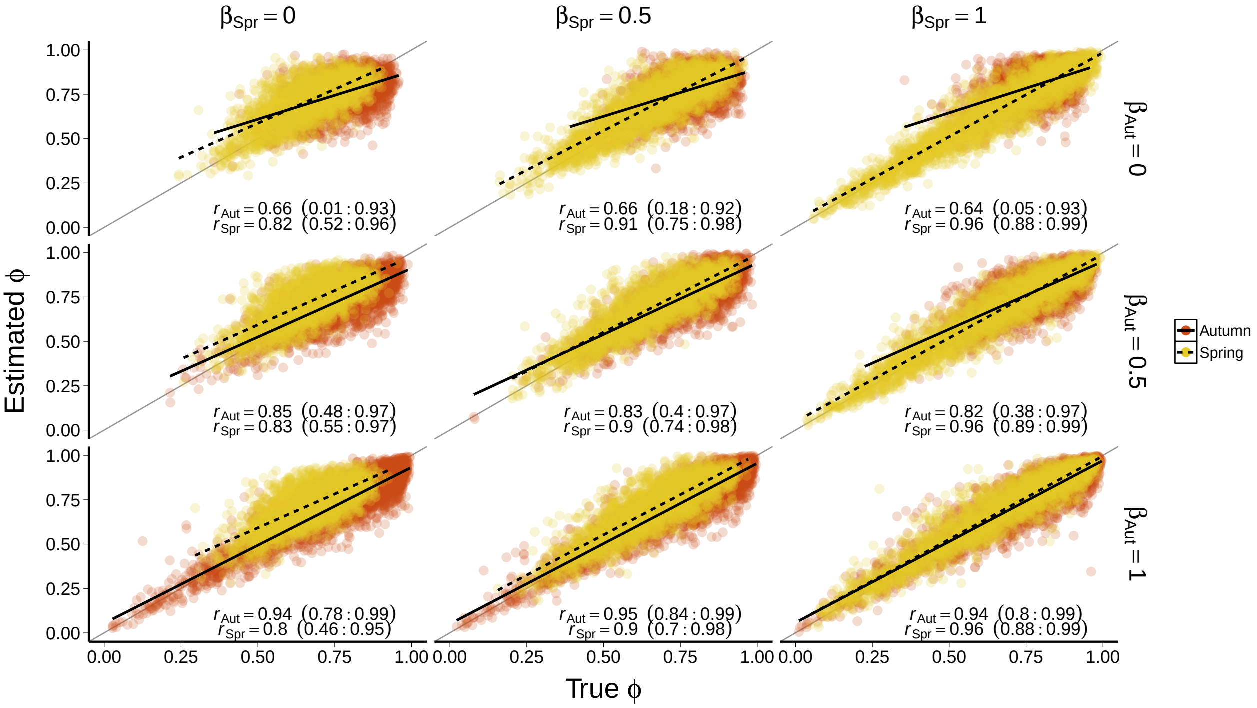

Figure S6: Correlation between estimated and true spring and autumn survival rates under the covariate models. For all simulations shown, $\sigma^2 = 0.25$. Points show estimates of $\phi_{j,t}$ from all 250 simulations in each scenario. Solid and dashed black lines show the mean correlation for each season and the solid gray line indicates 1:1 correspondance between estimated and true survival. Values in parentheses are the 95% credible interval of the r estimates.
